# The E3 Ubiquitin Ligase Activity of Trip12 Is Essential for Mouse Embryogenesis

**DOI:** 10.1371/journal.pone.0025871

**Published:** 2011-10-18

**Authors:** Masashi Kajiro, Mai Tsuchiya, Yoh-ichi Kawabe, Ryohei Furumai, Naoya Iwasaki, Yuki Hayashi, Miyuki Katano, Yuka Nakajima, Natsuka Goto, Tatsuya Watanabe, Akiko Murayama, Hisashi Oishi, Masatsugu Ema, Satoru Takahashi, Hiroyuki Kishimoto, Junn Yanagisawa

**Affiliations:** 1 Graduate School of Life and Environmental Sciences, University of Tsukuba, Tsukuba Science City, Ibaraki, Japan; 2 Center for Tsukuba Advanced Research Alliance, University of Tsukuba, Tsukuba Science City, Ibaraki, Japan; 3 Department of Anatomy and Embryology, Institute of Basic Medical Sciences, Graduate School of Comprehensive Human Sciences, University of Tsukuba, Tennoudai, Tsukuba, Japan; VIB & Katholieke Universiteit Leuven, Belgium

## Abstract

Protein ubiquitination is a post-translational protein modification that regulates many biological conditions [1], [2], [3], [4]. Trip12 is a HECT-type E3 ubiquitin ligase that ubiquitinates ARF and APP-BP1 [5], [6]. However, the significance of Trip12 *in vivo* is largely unknown. Here we show that the ubiquitin ligase activity of Trip12 is indispensable for mouse embryogenesis. A homozygous mutation in *Trip12* (*Trip12^mt/mt^*) that disrupts the ubiquitin ligase activity resulted in embryonic lethality in the middle stage of development. *Trip12^mt/mt^* embryos exhibited growth arrest and increased expression of the negative cell cycle regulator *p16* [7], [8], [9], [10]. In contrast, *Trip12^mt/mt^* ES cells were viable. They had decreased proliferation, but maintained both the undifferentiated state and the ability to differentiate. *Trip12^mt/mt^* ES cells had increased levels of the BAF57 protein (a component of the SWI/SNF chromatin remodeling complex) and altered gene expression patterns. These data suggest that Trip12 is involved in global gene expression and plays an important role in mouse development.

## Introduction

Protein ubiquitination plays an important role in various cellular events [1], [2], [3], [4]. Ubiquitination occurs through sequential steps that are catalyzed by activating (E1), conjugating (E2), and ligase (E3) enzymes. In this reaction, the specificity for distinct substrates is determined by the ubiquitin ligase enzymes. From a mechanistic standpoint, ubiquitin ligases fall into two groups: HECT domain E3s and RING domain E3s [11], [12], [13]. The HECT family contains a diverse N-terminal region and a conserved C-terminal HECT domain, which has a reactive Cys residue. HECT-type ubiquitin ligases form a thioester intermediate with ubiquitin at the conserved Cys residue before transferring ubiquitin to the target proteins [12], [13].

*Trip12* encodes a HECT domain containing an E3 ubiquitin ligase, which was originally identified as a thyroid hormone receptor-interacting protein in a yeast two-hybrid screen [14]. Recent reports indicate that Trip12 ubiquitinates ARF and APP-BP1 and regulates BAF57 turnover [5], [6], [15]. In this study, we showed that the ubiquitin ligase activity of Trip12 is essential for mouse development. We generated mice in which the coding region of the HECT domain within Trip12 was mutated (*Trip12^mt/mt^* mice). Targeted inactivation of Trip12 resulted in abnormal *p16* induction and embryonic lethality in mice. We also found that *Trip12^mt/mt^* ES cells were viable but had decreased cell cycle progression. *Trip12^mt/mt^* ES cells had accumulated levels of the BAF57 protein and different gene expression patterns. Our data suggest that Trip12 is involved in global gene expression and is essential for mouse development.

## Results

### Generation of Trip12 mutant mice

**Figure 1:**
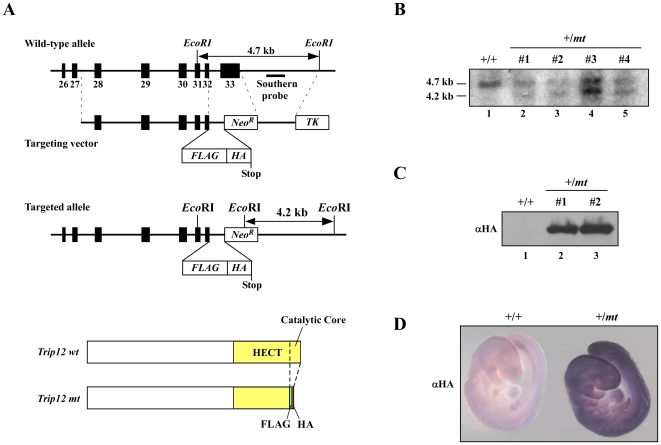
Generation of Trip12 mutant mice. (A) Schematic representation of the *Trip12* gene targeting construct. Exon 33 of *Trip12* genomic DNA containing the Cys residue that is required for ubiquitination was replaced in frame with FLAG and HA epitope tag sequences. Thus, the Trip12 mutant protein has FLAG and HA epitope tags instead of ubiquitination activity. Solid rectangles represent the exons of the *Trip12* gene. (B) Southern blot analysis of the targeted allele in heterozygote Trip12 mutant ES cells. (C) Western blot analysis of Trip12 mutant protein expression in ES cells. (D) Whole mount immunohistochemistry of wild-type and heterozygous *Trip12* embryos using an anti-HA antibody.

To study the function of Trip12 *in vivo*, we generated Trip12 mutant mice by replacing exon 33 with FLAG and HA epitope tag sequences in frame and a neomycin resistant gene. This mutant Trip12 lacks the catalytic core for the ubiquitin ligase activity and has FLAG and HA epitope sequences at the C-terminus (Fig. 1A). The targeting vector was linearized, electroporated into ES cells and ES cells were grown under G418 selection. Selected ES cell clones were verified by Southern blot analysis and several clones were correctly targeted (Fig. 1B). We also detected the mutant Trip12 proteins in the targeted clones by Western blotting using an anti-HA antibody (Fig. 1C). Heterozygous *Trip12^+/mt^* mice were viable and fertile and did not exhibit any overt phenotype. To elucidate the Trip12 expression pattern in mouse embryos, anti-HA staining was performed. As shown in Fig. 1D, Trip12 is ubiquitously expressed in the embryo at E9.5 (Fig. 1D).

### TRIP12 ubiquitin ligase activity is essential for mouse embryogenesis

*Trip12^+/mt^* mice were intercrossed in order to produce *Trip12^mt/mt^* mice. However, no *Trip12^mt/mt^* mice were generated, suggesting that the ubiquitin ligase activity of Trip12 is essential for mouse development. To characterize the timing of this embryonic lethality, embryos derived from the *Trip12^+/mt^* intercross were genotyped at different stages of gestation. *Trip12^+/+^*, *Trip12^+/mt^*, and *Trip12^mt/mt^* embryos were born at the expected Mendelian ratio and were viable up to E8.5, while no *Trip12^mt/mt^* embryos were observed between E11.5 and E13.5 (Table 1). From these results, we concluded that Trip12 plays an important role in mouse embryogenesis.

**Table 1:** Genotypes of the offspring from Trip12^+/*mt*^ intercrosses.

	No. of mice with the following					
	genotype:					
Age	+/+	+/mt	mt/mt	Absorbed	Empty	Total
E7.5	7 (23.3%)	17 (56.6%)	6 (20.0%)	0	0	30
E8.5	3 (27.3%)	6 (54.5%)	2 (18.2%)	0	0	11
E9.5	15 (31.3%)	23 (47.9%)	8 (16.7%)	0	2 (4.1%)	48
E10.5	18 (32.7%)	30 (54.5%)	6 (10.9%)	0	1 (1.8%)	55
E11.5	9 (27.3%)	15 (45.5%)	0	9 (27.3%)	0	33
E12.5	2 (28.6%)	2 (28.6%)	0	3 (42.9%)	0	7
E13.5	4 (26.7%)	9 (60.0%)	0	2 (13.3%)	0	15
Newborn	4 (30.8%)	9 (69.2%)	0	-	-	13
3 weeks	17 (36.2%)	30 (63.8%)	0	-	-	47

Percentage shows the ratio of each genotype in total number of embryos in each developmental stages.

### Phenotypes of *Trip12^mt/mt^* embryos

**Figure 2:**
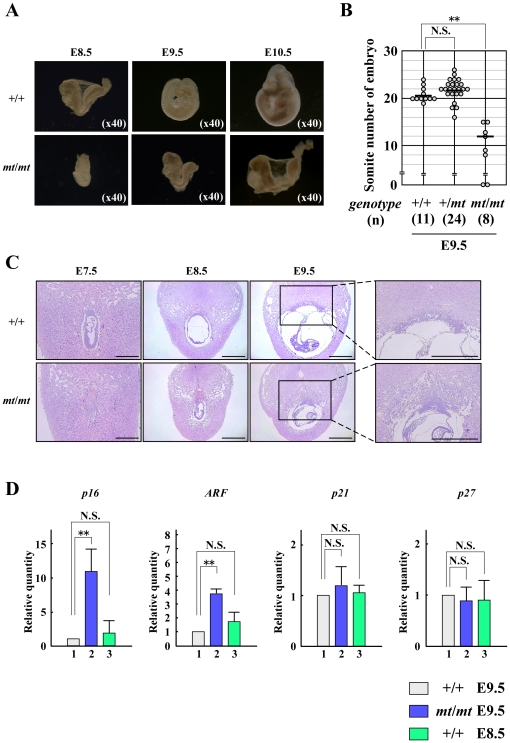
Analysis of wild-type (+/+) and *Trip12* mutant (*mt*/*mt*) embryos. (A) Different developmental stages (E8.5 to E10.5) of wild-type (+/+) and *Trip12* mutant (*mt*/*mt*) embryos. (×magnification) (B) The somite number of *Trip12^+/+^*, *Trip12^+/mt^*, and *Trip12^mt/mt^* embryos. The somite number of each embryo at E9.5 was scored by stereo microscopy. **, *p*<0.001; N.S., not significant. (C) Hematoxylin and eosin staining of sections of wild-type (+/+) and *Trip12* mutant (*mt*/*mt*) embryos at different developmental stages (E7.5 to E9.5). Placental tissues at E9.5 are enlarged in the right panel. Scale bars represent 1 mm. (D) The expression levels of negative cell cycle regulators in wild-type (+/+) and *Trip12* mutant (*mt*/*mt*) embryos. Total RNA was isolated from embryos, and the gene expression profiles were analyzed using real-time RT-PCR. Data are presented as the mean+s.d., n = 3. **, *p*<0.001; N.S., not significant.

To characterize the nature of this embryonic lethality, we studied the morphology of embryos from timed *Trip12^+/mt^* intercrosses at different stages of gestation. An examination of these embryos at various developmental stages revealed that the development of *Trip12^mt/mt^* embryos appeared to be delayed at E8.5 (Fig. 2A). Despite this developmental delay, there were no gross morphological abnormalities in *Trip12^mt/mt^* embryos. We also confirmed that the number of somites was decreased in *Trip12^mt/mt^* embryos compared to wild-type embryos at E9.5 (Fig. 2B).

The *Trip12^mt/mt^* embryos were further characterized histologically at different developmental stages. In addition to a developmental delay, *Trip12^mt/mt^* embryos had abnormal placenta formation (Fig. 2C). Normal labyrinth formation was not observed and a number of erythrocytes accumulated in the placenta of *Trip12^mt/mt^* embryos (Fig. 2C right panel). This phenotype might be caused by impaired development of the allantois [16].

Given Trip12 enzymatic activity which is involved in ARF regulation (4), we examined whether mutant show altered expression of ARF and related proteins which could explain the developmental arrest of *Trip12^mt/mt^* embryos. Therefore, we examined the expression levels of several cell cycle-related genes and found that *p16* and *ARF* expression levels were increased in *Trip12^mt/mt^* embryos (Fig. 2D). p16 is a cyclin-dependent kinase inhibitor and ARF increases p53 protein stability and promotes p53-dependent cell cycle arrest [7], [8], [9], [10]. However, the expression level of *p21*, a p53 target gene, was virtually unchanged. In addition, mutant embryo didn't show increased apoptosis compared to wild type embryo at corresponding developmental stage (Fig. S1). These data indicate that the embryonic lethality of *Trip12^mt/mt^* mice may be caused by abnormal *p16* induction rather than p53 activation.

### Generation and characterization of *Trip12^mt/mt^* ES cells

**Figure 3:**
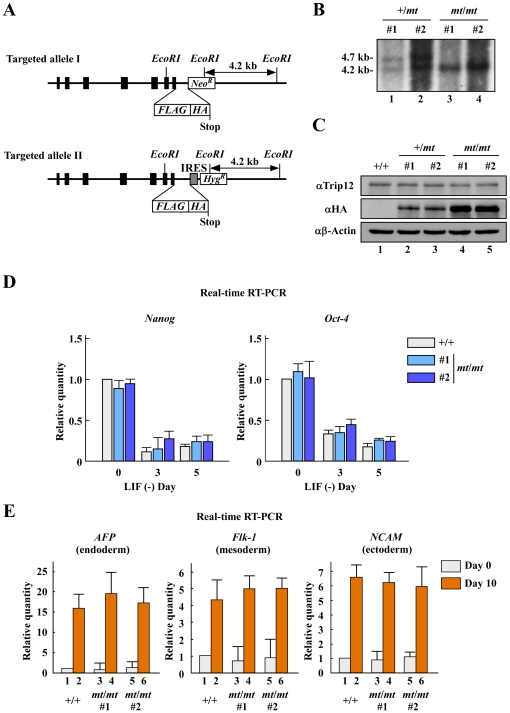
Generation and characterization of *Trip12* mutant (*mt*/*mt*) ES cells. (A) Schematic representation of the *Trip12* gene targeting construct (Targeted allele II). Solid rectangles represent the exons of the *Trip12* gene. (B) Southern blot analysis of the targeted allele in Trip12 mutant (*mt*/*mt*) ES cells. (C) Western blot analysis of Trip12 mutant protein expression in ES cells. (D) Expression levels of *Nanog* and *Oct-4* in Trip12 mutant (*mt*/*mt*) ES cells with or without LIF. The cells were cultured without LIF and harvested at the indicated time points. Total RNA was isolated from ES cells, and the gene expression profiles were analyzed using real-time RT-PCR. Data are presented as the mean+s.d., n = 3. (E) Embryoid body (EB) formation of wild-type (+/+) or Trip12 mutant (*mt*/*mt*) ES cells. The cells were cultured in suspension in ES cell medium without LIF in 10-mm dishes for 10 days. Scale bars: 100 µm. Total RNA was isolated from the cells, and the gene expression profiles were analyzed using real-time RT-PCR. Data are presented as the mean+s.d., n = 3.

To further investigate the function of Trip12, we generated two *Trip12^mt/mt^* ES cell clones [17 and #2] (Fig. 3A, 3B, and 3C). We examined the ability of *Trip12^mt/mt^* ES cells to self-renew and differentiate. The expression levels of *Nanog* and *Oct-4* were comparable in the two Trip12*^mt^* ^/*mt*^ ES cell clones and wild-type cell cultures with or without LIF (Fig. 3D). To assess whether ES cell differentiation was affected, we compared embryoid body (EB) formation on days 0 and 10. *Trip12^mt/mt^* ES cells displayed the same EB morphology (data not shown), and the expression patterns of endodermal (AFP)-, mesodermal (Flk-1)-, and ectodermal (NCAM)-specific genes were also similar between wild-type and *Trip12^mt/mt^* ES cells (Fig. 3E). These results suggest that Trip12 has a minor role in ES cell differentiation.

### *Trip12^mt/mt^* ES cells exhibit cell cycle defects

**Figure 4:**
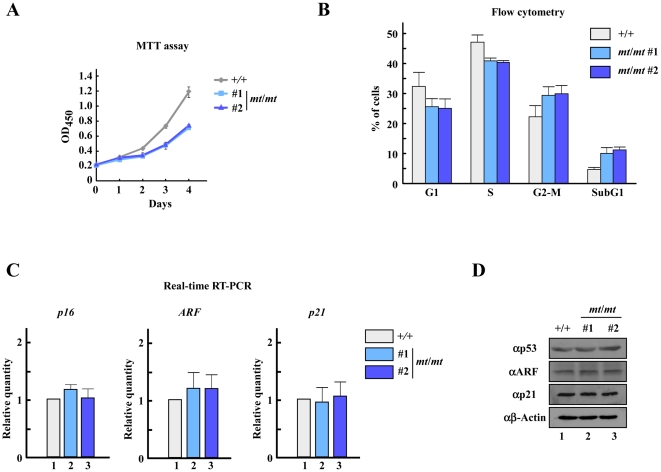
Cell cycle defects in *Trip12* mutant (*mt*/*mt*) ES cells. (A) Proliferation of Trip12 mutant (*mt*/*mt*) ES cells. The growth rate of wild-type or Trip12 mutant (*mt*/*mt*) ES cells was measured by the MTT assay. (B) Cell cycle progression in *Trip12* mutant (*mt*/*mt*) ES cells was analyzed by flow cytometry. (C) The expression levels of negative cell cycle regulators in wild-type (+/+) or *Trip12* mutant (*mt*/*mt*) ES cells. Total RNA was isolated from the cells, and the gene expression profiles were analyzed using real-time RT-PCR. Data are presented as the mean+s.d., n = 3. (D) Western blot analysis of proteins in the p53 pathway in wild-type (+/+) or Trip12 mutant (*mt*/*mt*) ES cells.

Next, we tested whether the Trip12 mutation leads to a defect in cell proliferation. The MTT assay showed that *Trip12^mt/mt^* ES cells had decreased cell growth (Fig. 4A). Therefore, we analyzed cell cycle progression by flow cytometry, and found that the G2-M and SubG1 populations were increased in *Trip12^mt/mt^* ES cells (Fig. 4B). We further examined the expression levels of *p16*, *ARF*, and *p21*. Unexpectedly, the expression levels of these genes were virtually unchanged in the cells (Fig. 4C). It was previously reported that Trip12 ubiquitinates ARF and inhibits p53-dependent transcription [6]. However, there were no detectable alterations in p53-related protein levels in *Trip12^mt/mt^* ES cells (Fig. 4D).

### Trip12 negatively regulates BAF57 protein stability and is involved in global gene expression in ES cells

**Figure 5:**
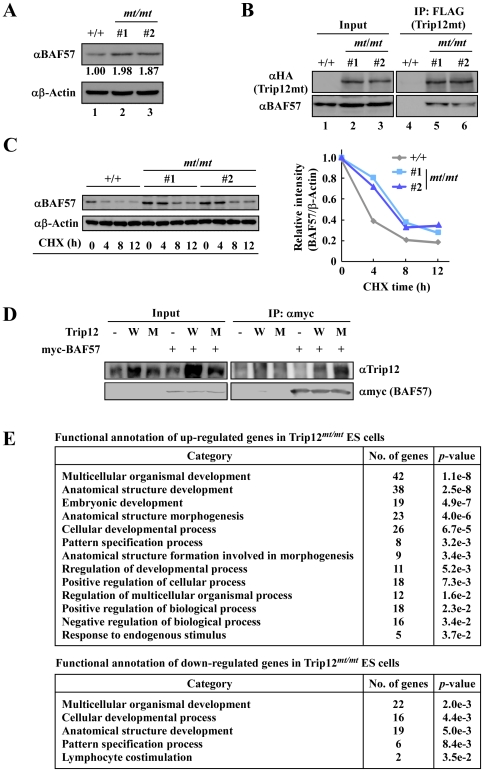
Loss of the ubiquitin ligase activity of Trip12 induces BAF57 protein accumulation in ES cells. (A) The BAF57 expression levels in wild-type (+/+) or Trip12 mutant (*mt*/*mt*) ES cells. The BAF57 protein levels were determined by immunoblotting, and the signal intensity was quantified. (B) Co-immunoprecipitation of Trip12 with BAF57. Trip12mt was immunoprecipitated from Trip12mt/mt ES cell lysate with an anti-FLAG antibody, and the immunoprecipitates were immunoblotted with the indicated antibodies. (C) Trip12 regulates the turnover of BAF57. The cells were treated with 100 µg/ml cycloheximide and lysed at various time points. The protein lysates were separated by SDS-PAGE and detected by immunoblotting. (D) Wild type or mutant Trip12 was ectopically expressed with or without myc-tagged BAF57. Myc-BAF57 was immunoprecipitated with anti-myc antibody and immunoblotted with anti-Trip12 or anti-myc antibody. (E) A comparison of the mouse whole-genome RNA expression profile between wild-type and Trip12 mutant (*mt*/*mt*) ES cells. DAVID and the annotation sources GOTERM_BP2 (Biological process) were used to identify the functional categories.

Recent reports also suggest that Trip12 regulates BAF57 protein turnover [15]. Therefore, we examined whether inactivation of the Trip12 ubiquitin ligase activity affects the stability of BAF57. As shown in Fig. 5A, the endogenous BAF57 protein levels were increased in *Trip12^mt/mt^* ES cells (Fig. 5A). We also confirmed that endogenous BAF57 interacts with Trip12mt from targeted allele in ES cells (Fig. 5B). In addition, cycloheximide experiments showed that the turnover rate of endogenous BAF57 was decreased in *Trip12^mt/mt^* ES cells (*t*_1/2_≫6 h) compared to that in wild-type cells (*t*_1/2_≈4 h) (Fig. 5C). As shown in Fig. 5D, ectopically expressed wild type and mutant Trip12 were interacting with GST-BAF57 and mutant Trip12 showed stronger interaction than wild type suggesting that loss of catalytic activity of Trip12 resulted in decreased dissociation of BAF57 from Trip12. These data demonstrate that the ubiquitin ligase activity of Trip12 is required for BAF57 turnover in ES cells. BAF57 is a component of the SWI/SNF chromatin remodeling complex and involved in a variety of cellular processes [18], [19], [20], [21], [22], [23], [24], [25], [26]. Therefore, a gene expression study using microarray analysis was performed to elucidate the underlying mechanism that leads to cell proliferation defects in *Trip12^mt/mt^* ES cells. Microarray analysis of variance identified a total of 7635 genes that were significantly expressed in wild-type and *Trip12^mt/mt^* ES cells (*p*-values<0.05). To identify statistically significant functional categories by gene ontology, we selected genes that had greater than 2-fold changes in expression and placed these genes into DAVID [27]. As shown in Fig. 5D, both up- and down-regulated genes were enriched in the developmental process (Fig. 5D). These data suggest that Trip12 regulates gene expression by destabilizing BAF57 during mouse development.

## Discussion

In this report, we have determined that the E3 ubiquitin ligase activity of Trip12 is indispensable for mouse development. Previous reports indicated that Trip12 promotes the ubiquitination and degradation of ARF [6]. The down-regulation of Trip12 expression stabilizes ARF and enhances ARF-dependent, p53-mediated cell cycle arrest in normal human fibroblasts [6]. However, in our experiments, the p53 pathway was not activated in either *Trip12^mt/mt^* mice or *Trip12^mt/mt^* ES cells. Although *Trip12^mt/mt^* mice had increased ARF expression levels, the p21 (which is a well known p53 target gene) expression levels were unchanged in these mice. These findings suggest that the growth arrest phenotype of *Trip12^mt/mt^* mice was caused by p16 expression rather than ARF induction. Previous reports also showed that Trip12-mediated ARF ubiquitination is inhibited by c-Myc [6]. It is known that c-Myc expression levels are higher in mouse embryos and ES cells compared to differentiated cells [17], [28], [29], [30]. Considering these results, we speculate that Trip12 minimally contributes to ARF ubiquitination during mice embryogenesis because of elevated c-Myc expression. On one hand, our experiments showed that BAF57 protein stability was regulated by Trip12-mediated ubiquitination and that the expression profiles of several genes were changed between wild-type and *Trip12^mt/mt^* ES cells. This finding suggests that the ubiquitination activity of Trip12 is tightly regulated by individual target proteins. Further experiments using conditional knockout approaches and the Trip12 protein complex that is purified from different cell types may help elucidate the exact *in vivo* functions of the Trip12 protein.

## Materials and Methods

### Cell culture and embryoid body (EB) formation

E14 ES cells (ATCC) were maintained on mouse embryo fibroblast (MEF) feeder cell layers in Dulbecco-modified Eagle medium containing 14% KnockOut Serum Replacement (Invirtogen), 1% fetal bovine serum, 100 units/ml LIF, 0.1 mM nonessential amino acids, 1 mM sodium pyruvate, 2 mM L-glutamine, and 100 µM 2-mercaptoethanol. For EB formation assays, 2×10^5^ cells were seeded into 35 mm low attachment sterile cell plate in 2 ml ES cell medium without LIF. Fresh medium was provided every two days.

### Targeting vectors and generation of Trip12 mutant mice

The targeting vectors for *Trip12* were constructed by inserting FLAG and HA epitope tag sequences in frame and Neo or IRES-hyg cassette into the exon 33 of the mouse *Trip12* genome. Correctly targeted ES cell clone were injected into C57BL/6 blastocysts, producing chimeric mice. Highly chimeric males were mated to females, and then the progeny were intercrossed. All experiments were performed in accordance with the Declaration of Helsinki and were approved by University of Tsukuba Ethics Committee for Animal Experiments (approval ID:040042).

### Western Blot

Cells were lysed in TNE buffer [10 mM Tris-HCl (pH 7.8), 1% NP-40, 150 mM NaCl, 1 mM EDTA, 1 µM phenylmethylsulfonyl fluoride, and 1 µg/ml aprotinin]. The proteins were separated by SDS-PAGE, transferred onto PVDF membranes (Millipore), immunoblotted with indicated antibodies. The antibodies employed in this study included mouse monoclonal antibodies specific for β-Actin (Sigma); rat monoclonal antibody for HA (Roche) and ARF (Calbiochem). Rabbit polyclonal antibody for BAF57 (GeneTex) and Trip12 (Bethyl Laboratories).

### Real-time RT-PCR

Embryos and cells were homogenized in 1 ml of sepasol (nacalai tesque) and total RNA was extracted according to the instruction manual. cDNA was synthesized from total RNA using RevatraAce reverse transcriptase (Toyobo) and random primers. Real-time PCRs were performed to amplify fragments representing for the indicated mRNA expression. We used primers 5′- CATCTGGAGCAGCATGGAGTC-3′ and 5′-GGGTACGACCGAAAGAGTTCG-3′ for *p16*, 5′-GTTCTTGGTCACTGTGAGGATTCAG-3′ and 5′-CCATCATCATCACCTGGTCCAG-3′ for *ARF*, 5′-TTGCACTCTGGTGTCTGAGC-3′ and 5′-TGCGCTTGGAGTGATAGAAA-3′ for *p21*, 5′-TTGGGTCTCAGGCAAACTCT-3′ and 5′-CTGTTGGCCCTTTTGTTTTG-3′ for *p27*, 5′-AGTACCTCAGCCTCCAGCAGAT-3′ and 5′-GCTTGCACTTCATCCTTTGGTT-3′ for *Nanog*, 5′-CACGAGTGGAAAGCAACTCA-3′ and 5′-TTCATGTCCTGGGACTCCTC-3′ for *Oct-4*, 5′-CATGAACAGGTTCATCTATG-3′ and 5′-GTTGTCAGCTTTGCAGCATG-3′ for *AFP*, 5′-TGTCTATGTTCGAGATTACAG-3′ and 5′-CATTGAGGTTTGAAATCGAC-3′ for *Flk-1*, 5′-GGTCATTGTGAATGTACCAC-3′ and 5′-CCTTTGTCCAGCTCATGGTG-3′ for *NCAM*,

### Cycloheximide treatment

Cells were treated with 100 µg/ml cycloheximide to prevent *de novo* protein synthesis, and lysed at various time points following cycloheximide treatment. The proteins were then separated by SDS-PAGE and detected by immunoblotting.

### Whole mount immunostaining

Whole mount immunostaining of embryos were performed as described previously [31].

### Microarray procedure and data analysis

The microarray procedure was performed as described in the Affymetrix GeneChip Expression Analysis manual (Affimetrix). Each array was analysed using GeneSpring (Agilent) program. Patterns were analysed to identify functional categories using Database for Annotation, Visualization and Integrated Discovery (DAVID) (http:// david.abcc.ncifcrf.gov) [27]. We used the functional annotation tool program and reported only GOTERM_BP (Biological process) that had *p*-values of <0.05.

### Statistical analysis

All the data are representative of at least three different experiments. Statistical analysis was performed using *t*-test.

## Supporting Information

Figure S1 **TUNEL staining of wild type E8.5 embryo and** ***Trip12^mt/mt^*** embryo at corresponding developmental stage.
(TIF)
