# Supplementary figures and images for: The E3 Ubiquitin Ligase Activity of Trip12 Is Essential for Mouse Embryogenesis
Source: PLoS One. 2011 Oct 18;6(10):e25871. doi: 10.1371/journal.pone.0025871 (PMC3196520; doi:10.1371/journal.pone.0025871)

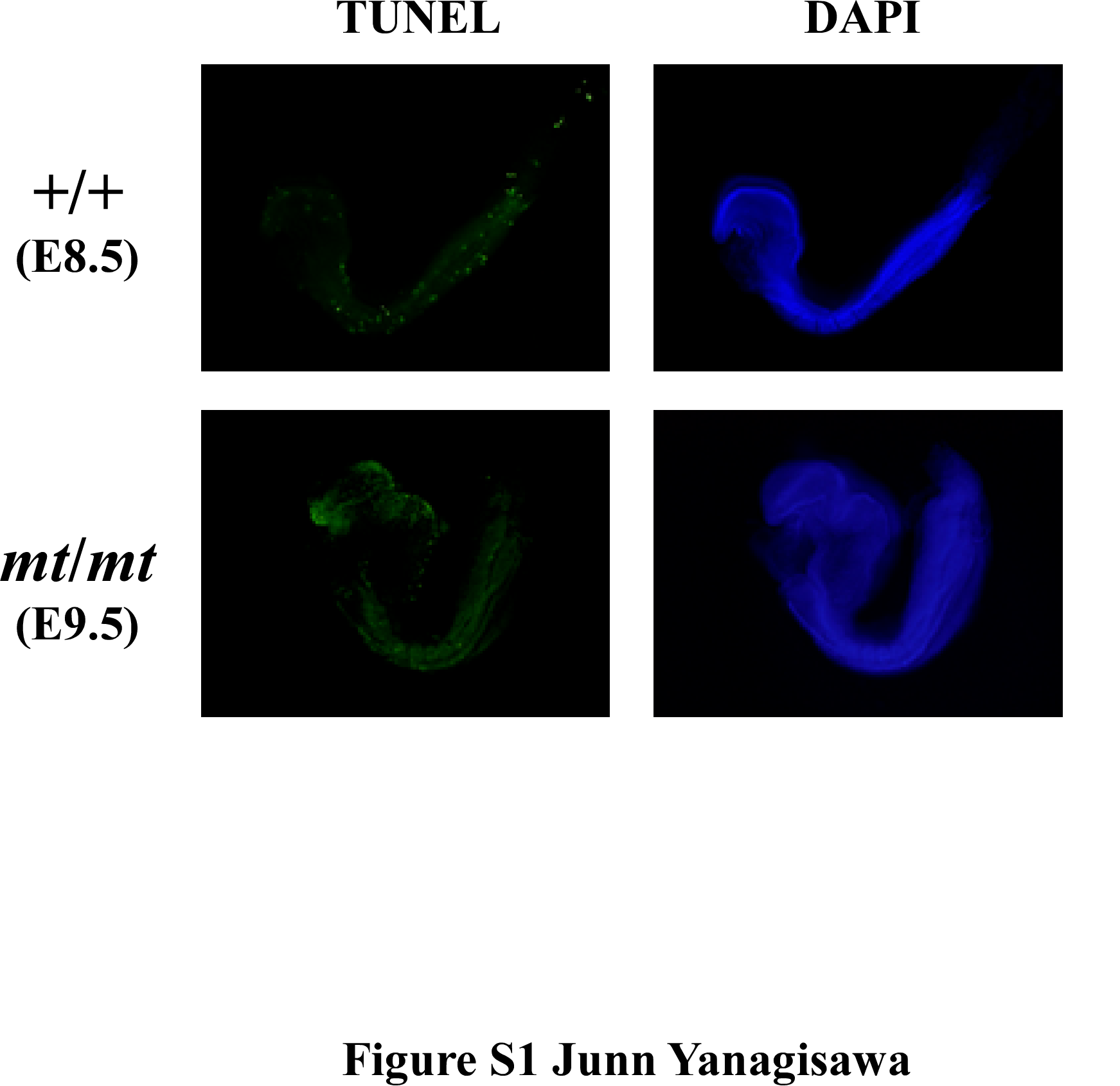

Supplement: Figure S1 — TUNEL staining of wild type E8.5 embryo and Trip12mt/mt embryo at corresponding developmental stage. (TIF) [file pone.0025871.s001.tif]
